# Keeping a Good Attitude: A Quaternion-Based Orientation Filter for IMUs and MARGs

**DOI:** 10.3390/s150819302

**Published:** 2015-08-06

**Authors:** Roberto G. Valenti, Ivan Dryanovski, Jizhong Xiao

**Affiliations:** 1 The City College of New York, The City University of New York, Convent Avenue and 140th Street, New York, NY 10031, USA; E-Mail: robertogl.valenti@gmail.com; 2 The Graduate Center, The City University of New York, 365 Fifth Avenue, New York, NY 10016, USA; E-Mail: ivan.dryanovski@gmail.com

**Keywords:** orientation estimation, inertial measurement unit, magnetic angular rate and gravity, quaternions, micro aerial vehicles

## Abstract

Orientation estimation using low cost sensors is an important task for Micro Aerial Vehicles (MAVs) in order to obtain a good feedback for the attitude controller. The challenges come from the low accuracy and noisy data of the MicroElectroMechanical System (MEMS) technology, which is the basis of modern, miniaturized inertial sensors. In this article, we describe a novel approach to obtain an estimation of the orientation in quaternion form from the observations of gravity and magnetic field. Our approach provides a quaternion estimation as the algebraic solution of a system from inertial/magnetic observations. We separate the problems of finding the “tilt” quaternion and the heading quaternion in two sub-parts of our system. This procedure is the key for avoiding the impact of the magnetic disturbances on the roll and pitch components of the orientation when the sensor is surrounded by unwanted magnetic flux. We demonstrate the validity of our method first analytically and then empirically using simulated data. We propose a novel complementary filter for MAVs that fuses together gyroscope data with accelerometer and magnetic field readings. The correction part of the filter is based on the method described above and works for both IMU (Inertial Measurement Unit) and MARG (Magnetic, Angular Rate, and Gravity) sensors. We evaluate the effectiveness of the filter and show that it significantly outperforms other common methods, using publicly available datasets with ground-truth data recorded during a real flight experiment of a micro quadrotor helicopter.

## 1. Introduction

The accurate estimation of the orientation of a rigid body, relative to an inertial frame, is required for a wide range of applications. For the purpose of navigation, such estimation has employed high precision inertial and magnetic sensors, but the recent development of low-cost and light-weight MicroElectroMechanical Systems (MEMS) has allowed smaller and cheaper inertial sensors to be adopted for a wider range of applications and even in the daily use of consumer electronics such as game consoles and mobile devices. In robotics, the evolution of Micro Aerial Vehicles (MAVs) has increased in the last decade, and research is moving toward their full autonomization. Navigation and control of MAVs are possible thanks to the MEMS-based Inertial Measurement Units (IMU) that meet the MAVs’ limited size and payload requirement.

Data provided by low-cost IMU is affected by high noise levels and time-varying biases. Therefore, a sensor-fusion algorithm must be used to process the data to obtain a smooth and bias-free estimation of the orientation maintaining a low computational cost for running on the onboard processor. The orientation can be generally represented in three principal forms: Euler angles, quaternion, and Direction Cosine Matrix (DCM). The orientation in Euler form is expressed using three angles; it is conceptually easy to understand, but may reach a singularity state commonly referred as “gimbal lock”. DCM and quaternion do not incur a singularity state but the DCM represents the orientation by a 3 × 3 matrix. Furthermore, the quaternion representation offers a linear formulation of the orientation dynamics.

In this article, we propose a deterministic approach to solve Whaba’s problem [1] given gravity and magnetic field observations provided by the MARG sensor. This method returns an estimation of the attitude in quaternion form without leading to ambiguous results and avoiding singularity problems. Moreover, it does not need a predefined knowledge of the magnetic field direction. We also propose a new approach to a complementary filter for fusing together gyroscope data with acceleration and magnetic field measurements from IMU and MARG sensors to obtain an orientation estimation in quaternion form. This algorithm is meant to be used onboard MAVs because of its low computational burden, robustness, and fast convergence. However, we believe our contribution is applicable beyond the field of MAV and aerial robotics.

This article is organized as follows. Section 2 explores the literature of attitude estimation methods previously proposed as solution to Whaba’s problem. Further, in the same section, we briefly survey several fusion algorithms that exploit the measurements given by gyroscope, accelerometer, and magnetometer of an IMU/MARG sensor, to obtain a more accurate evaluation of the orientation. Section 3 briefly provides an overview of some properties of unit quaternions and how they can be used to define rotations in the 3D space. Section 4 analyzes the novel approach taken to obtain the quaternion orientation of a rigid body from earth-field observations as a closed-form solution. Section 5 explains the novel quaternion-based complementary filter. Section 6 is dedicated to the experiments and results interpretations, and Section 7 concludes the article by summarizing the findings.

## 2. Previous Work

The problem of finding the optimal attitude estimation, given multiple unit vector correspondences across two frames, was formulated by Whaba in 1965 [1]. Whaba’s problem was meant to estimate the attitude of a satellite by finding the optimal orthogonal matrix (the attitude matrix) that minimizes a least-square loss function using *n* numbers of unit vectors pairs where n≥2. Many algorithmic solutions of Whaba’s problem have been proposed. They are generally classified into two categories: deterministic and optimal, according to the popular definition by Wertz [2]. Deterministic algorithms use a minimal set of data and derive the attitude by solving nonlinear equations, whereas optimal algorithms use more than a minimal set of measurements and compute the attitude by minimizing an appropriate cost function.

A very well known deterministic algorithm is the three axis attitude determination (TRIAD) [3]. It constructs two triads of orthonormal unit vectors by combining the normalized measurement of two nonparallel reference vectors and provides an estimation of the attitude matrix. QUaternion ESTimator (QUEST) [3] is a famous optimal algorithm that produces the attitude estimation in quaternion form given a set of 3D reference unit vectors in a fixed frame and their corresponding observations in the local frame. It is based on Davenport’s q-method [4] that finds the optimal quaternion by minimizing a quadratic gain, which is a transformation of Whaba’s loss function through the parametrization of the orientation matrix by the unit quaternion. Many other techniques have been proposed, such as Singular Value decomposition (SVD) [5], Polar Decomposition (PD) [6], Euler-n [7], Fast Optimal Matrix Algorithm (FOAM) [8], and Energy Approach Algorithm (EAA) [9]. All of them produce an optimal attitude estimation and they differ from each other in their computational speed. A complete survey and analysis of attitude estimation methods in quaternion form using vector observation is provided by Markley and Mortari in [10].

It is well known that in applications based on inertial/magnetic sensors, to estimate the attitude, the two local observations of the earth’s fields (gravity and local magnetic field) are compared to the reference vectors that are supposed to be fixed. This assumption can lead to problems when the local magnetic field is perturbed by ferromagnetic objects or electrical appliances. In these cases, when algorithms such as QUEST are employed, the attitude estimation would be subject to errors not only in the yaw component but also in the pitch and roll. To avoid this problem, Yun *et al*. [11] proposed the Factored Quaternion Algorithm (FQA), which computes the orientation of a rigid body based on earth gravity and magnetic field measurements. The quaternion orientation is estimated by analyzing a series of three sequential rotations. This approach allows to reduce the orientation error, caused by the presence of local magnetic disturbances, only into the error in the yaw component maintaining the accuracy of the QUEST algorithm. To obtain the quaternion orientation from gravity and magnetic field observations, the method presented in this article solves the problem described above by separating the quaternion in only two parts, one for the roll and pitch components and one for the yaw. Both quaternions are found as an algebraic solution of a system instead of the result of an optimization problem. As inertial sensors provide at most the observation of 2 pairs of vectors (given a priori the global-frame gravity and magnetic field vectors), the use of optimal algorithms does not produce any improvement but only reduces the convergence speed of the orientation.

In the class of deterministic methods, Euler-2 by Daniele Mortari [7] is a mathematical approach to compute the Euler axis whose application is restricted when only two unit-vector pairs are available. Overall, besides the TRIAD algorithm, deterministic attitude-determination algorithms are not very frequent and their study is not extensive. Nonetheless, in IMU applications, only two sets of vector observations are provided, thus even the optimal approaches would have the same level of accuracy as a deterministic method.

To obtain a better estimation of the orientation, acceleration and magnetic field data are fused together with angular rate readings from a gyroscope. Although many approaches have been adopted for filtering gyroscope data with inertial measurements, the most commonly used techniques are Extended Kalman filtering (EKF) and complementary filters. A survey of other nonlinear attitude estimation methods can be found in [12].

Kalman filtering based techniques adopt a probabilistic determination of the state modeled as a Gaussian distribution given the system’s model. They are widely used in aerospace applications [13,14], human motion analysis [15,16,17] and robotics [18,19].

A Complementary filter is a common alternative to the EKF because of its simplicity and effectiveness. It uses an analysis in the frequency domain to filter the signals and combine them together to obtain an estimation of the orientation without any statistical description. In UAV applications, the use of complementary filters [20,21,22] is often preferred to the EKF because EKFs can be complicated to implement and the convergence is slower because of the time required for the linear regression iterations.

Most of the recent sensor fusion algorithms for inertial/magnetic sensors provide orientation estimation in quaternion form. Quaternions are a useful mathematical tool that require less computation time because of their minimal number of parameters and do not result in singularity configurations as the Euler representation does. Further, rotations of vectors are simply performed by quaternion multiplications.

Marins *et al*. [23] propose two different Kalman filter approaches to estimate the orientation in quaternion form from a MARG sensor. Both methods have the same 7-state (angular velocities and quaternion) process model but different measurement models. The first measurement model uses each MARG output in a 9-element measurement vector resulting in a complicated EKF. The second approach uses an external Gauss-Newton algorithm to directly estimate the quaternion measurement that will be part, along with the angular velocities, of the measurement vector. In this case, the relation between the process and measurement model is linear allowing the use of a simpler Kalman Filter. The quaternion-based EKF presented by Sabatini [16] is similar to the first approach of Marins *et al*. [23], but the state is composed by the unit quaternion augmented with accelerometer and magnetometer bias vectors for on-line calibration. In [24] Sabatini presents a similar EKF where the state is augmented with magnetic distortions vector, modeled as a Gauss-Markov process, to reduce the heading drift in magnetically non-homogeneous environments. Choukroun *et al*. [14] present a novel linear pseudo-measurement model that combined with the linear measurement model, yields a linear Kalman Filter algorithm that eliminates the linearization procedure of an EKF. Bachman *et al*. [25] present an efficient quaternion-based complementary filter for human-body-motion tracking.

Euston *et al*. [21] present a nonlinear quaternion-based complementary filter to estimate the attitude of a UAV given measurement from a low-cost IMU. The filter is augmented by a first-order model of the vehicle dynamics to compensate for external centripetal acceleration. In the work by Madgwick *et al*. [26], a constant gain filter is adopted to estimate the attitude in quaternion form of a rigid body by using data from a MARG sensor. A first quaternion estimation is obtained by gyroscope output integration and then it is corrected by a quaternion from the accelerometer and magnetometer data computed through a gradient descent algorithm. Madgwick’s method ensures good attitude estimation at low computational cost. Further, it addresses the problem of the local magnetic disturbances that, when present, affect all the orientation components (roll, pitch, and yaw). By reducing the constraint of the magnetic field vector rotation, it is able to limit the effect of the magnetic disturbances to only affect the yaw component of the orientation. The last two constant gain filters, by Euston *et al*. [21], and by Madgwick *et al*. [26], are commonly used because they offer good performances at low computational cost and a comparative analysis of the two algorithms is presented in [27]. The adaptive-gain complementary filter proposed by Tian *et al*. [28] fuses a quaternion estimation from fast moving gyroscope signal with a quaternion, from slow moving accelerometer and magnetometer signals, computed through a Gauss-Newton algorithm. For more robust results, the gain is adaptively adjusted according to the convergence rate of the low-frequency estimation and the divergence rate of the high-frequency estimation. Fourati *et al*. [29] combine the output of a Levenberg-Marquardt algorithm, the inputs for which are acceleration and local magnetic field measurements, with the angular rate measurements in a complementary observer based on the multiplicative correction technique.

To improve the performance of rigid body orientation estimation from low cost IMU/MARG under dynamic motion, different approaches can be adopted. A simple switching method, as the one proposed in the complementary filter presented in [30], can be used to determine the gain value under varying dynamics sensed by the accelerometer. Another common method includes an adaptive measurement noise covariance matrix that, in a Kalman filter framework, can be tuned adaptively to yield optimal performance during the dynamic periods as proved in [31,32]. Alternatively, the acceleration can be modeled to directly estimate the external acceleration and thus used to reduce the attitude error, as in the Kalman filter proposed in [33].

The quaternion-based complementary filter proposed in this article can be used for both IMU and MARG sensors. It ensures fast convergence and robustness thanks to the analytical derivation of the correction quaternion. We address the problem of the magnetic disturbances by separating the quaternion corrections in two different correction sequences and adopting the method presented in [26] that also makes the algorithm independent on external parameters. Further, we adopt an adaptive-gain approach to reduce the estimation error during high dynamic motion.

## 3. Background Theory

Any arbitrary orientation in the 3D space of a frame *A* with respect to a frame *B* can be represented by a unit quaternion ${}_{\text{A}}^{\text{B}}q$ defined as following:

(1) $${}_{\text{A}}^{\text{B}}q={\left[\begin{array}{cccc} q_{0} & q_{1} & q_{2} & q_{3} \end{array}\right]}^{T}={\left[\begin{array}{cccc} \cos\frac{\alpha }{2} & e_{x}\sin\frac{\alpha }{2} & e_{y}\sin\frac{\alpha }{2} & e_{z}\sin\frac{\alpha }{2} \end{array}\right]}^{T}$$

where α is the rotation angle and *e* is the unit vector that represents the rotation axis. The quaternion conjugate of ${}_{\text{A}}^{\text{B}}q$, given its unit norm, is equivalent to the inverse quaternion and describes the inverse rotation. Therefore, the conjugate quaternion can be used to represent the orientation of frame *B* relative to frame *A*, as defined below.

(2) $${{}_{\text{A}}^{\text{B}}q}^{*}={}_{\text{B}}^{\text{A}}q={\left[\begin{array}{cccc} q_{0} & -q_{1} & -q_{2} & -q_{3} \end{array}\right]}^{T}$$

The orientation quaternion after a sequence of rotations can be easily found by quaternion multiplication where each quaternion represents the orientation of a frame with respect to the rotated one. For example, given three frames *A*, *B* and *C*, and given the quaternion ${}_{\text{A}}^{\text{B}}q$ orientation of frame *A* expressed with respect to frame *B* and given the quaternion ${}_{\text{B}}^{\text{C}}q$ orientation of frame *B* expressed with respect to frame *C*, the orientation of frame *A* with respect to frame *C* is characterized by:

(3) $${}_{\text{A}}^{\text{C}}q={}_{\text{B}}^{\text{C}}q⊗{}_{\text{A}}^{\text{B}}q$$

where quaternion multiplication, given two quaternions ***p*** and ***q***, is defined as

(4) $$p⊗q=\left[\begin{array}{c} p_{0}q_{0}-p_{1}q_{1}-p_{2}q_{2}-p_{3}q_{3}\\ p_{0}q_{1}+p_{1}q_{0}+p_{2}q_{3}-p_{3}q_{2}\\ p_{0}q_{2}-p_{1}q_{3}+p_{2}q_{0}+p_{3}q_{1}\\ p_{0}q_{3}+p_{1}q_{2}-p_{2}q_{1}+p_{3}q_{0} \end{array}\right]$$

Unit quaternions can be applied to operate rotations of 3D vectors. For example a vector *^A^v*, expressed with respect to the *A* frame, can be expressed with respect to the *B* frame by the following operation:

(5) $${{}_{}^{\text{B}}v}_{q}={}_{\text{A}}^{\text{B}}q⊗{{}_{}^{\text{A}}v}_{q}⊗{{}_{\text{A}}^{\text{B}}q}^{*}$$

where the symbol ⊗ indicates the quaternion multiplication, and *^A^v_q_* and *^B^v_q_* are the observations of the vector *v*, in the two reference frames, written as pure quaternions as shown in Equation (6).

(6) $$v_{q}={\left[\begin{array}{cc} 0 & v \end{array}\right]}^{T}={\left[\begin{array}{cccc} 0 & v_{x} & v_{y} & v_{z} \end{array}\right]}^{T}$$

The inverse rotation that describes the vector *^B^v* relative to the frame *A* can be easily found by using the property of the conjugate quaternion and it is presented in Equation (7).

(7) $${{}_{}^{\text{A}}v}_{q}={{}_{\text{A}}^{\text{B}}q}^{*}⊗{{}_{}^{\text{B}}v}_{q}⊗{}_{\text{A}}^{\text{B}}q={}_{\text{B}}^{\text{A}}q⊗{{}_{}^{\text{B}}v}_{q}⊗{{}_{\text{B}}^{\text{A}}q}^{*}$$

The rotation defined in Equation (5) can be written in matrix form as in Equation (8)

(8) $${}_{}^{\text{B}}v=R\;\left({}_{\text{A}}^{\text{B}}q\right){}_{}^{\text{A}}v$$

where $R\;\left({}_{\text{A}}^{\text{B}}q\right)$, which belongs to the 3D special orthogonal group SO(3), is the direct cosine matrix (DCM) given in terms of the orientation quaternion ${}_{\text{A}}^{\text{B}}q$ as shown below.

(9) $$R\;\left({}_{\text{A}}^{\text{B}}q\right)=\left[\begin{array}{ccc} q_{0}^{2}+q_{1}^{2}-q_{2}^{2}-q_{3}^{2} & 2(q_{1}q_{2}-q_{0}q_{3}) & 2(q_{1}q_{3}+q_{0}q_{2})\\ 2(q_{1}q_{2}+q_{0}q_{3}) & q_{0}^{2}-q_{1}^{2}+q_{2}^{2}-q_{3}^{2} & 2(q_{2}q_{3}-q_{0}q_{1})\\ 2(q_{1}q_{3}-q_{0}q_{2}) & 2(q_{2}q_{3}+q_{0}q_{1}) & q_{0}^{2}-q_{1}^{2}-q_{2}^{2}+q_{3}^{2} \end{array}\right]$$

Given the properties of the elements of the SO(3), the inverse rotation can be defined as:

(10) $${}_{}^{\text{A}}v=R\;\left({}_{\text{B}}^{\text{A}}q\right){}_{}^{\text{B}}v=R^{T}\;\left({}_{\text{A}}^{\text{B}}q\right){}_{}^{\text{B}}v$$

## 4. Quaternion from Earth-Field Observations

In this section, we analyze the algebraic derivation of a quaternion from the observation of the earth’s fields. For a clear understanding of the following derivation, let us first define a notation that will be used throughout this article. We refer to the local (sensor) frame as *L*, and the global (earth) frame as *G*. We can define the measured acceleration ${}_{}^{\text{L}}\text{A}$ and the true earth gravitational acceleration ${}_{}^{\text{G}}g$ as the unit vectors:

$$\begin{array}{ccc} {}_{}^{\text{L}}a & ={\left[\begin{array}{ccc} {\text{A}}_{x} & {\text{A}}_{y} & {\text{A}}_{z} \end{array}\right]}^{T}, & ∥a∥=1\\ {}_{}^{\text{G}}g & ={\left[\begin{array}{ccc} 0 & 0 & 1 \end{array}\right]}^{T} \end{array}$$

Similarly, we define the measured local magnetic field ${}_{}^{\text{L}}m$ and the true magnetic field ${}_{}^{\text{G}}h$ as the unit vectors:

$$\begin{array}{ccc} {}_{}^{\text{L}}m & ={\left[\begin{array}{ccc} m_{x} & m_{y} & m_{z} \end{array}\right]}^{T}, & ∥m∥=1\\ {}_{}^{\text{G}}h & ={\left[\begin{array}{ccc} h_{x} & h_{y} & h_{z} \end{array}\right]}^{T}, & ∥h∥=1 \end{array}$$

Finally, the gyroscopes measure the angular velocity ${}_{}^{\text{L}}\omega$ around the three sensor frame axes:

$${}_{}^{\text{L}}\omega ={\left[\begin{array}{ccc} \omega_{x} & \omega_{y} & \omega_{z} \end{array}\right]}^{T}$$

Note that most IMUs usually measure a non-normalized vector *a* and *m*. However, for the purposes of derivation in this article, we assume that the quantities have been normalized. The only relevant units are those of ω, which we assume are radians per second.

We present an algebraic derivation of the orientation quaternion ${}_{\text{G}}^{\text{L}}q$, of the global frame (G) relative to the local frame (L), as a function of ${}_{}^{\text{L}}\text{A}$ and ${}_{}^{\text{L}}m$. We have two independent sensors observing two independent fields; a straightforward way to formulate the quaternion is through the inverse orientation which rotates the measured quantities ${}_{}^{\text{L}}\text{A}$ and ${}_{}^{\text{L}}m$ into the reference quantities ${}_{}^{\text{G}}\text{G}$ and ${}_{}^{\text{G}}h$:

(11) $$\left\{\begin{array}{c} R^{T}\;\left({}_{\text{G}}^{\text{L}}q\right){}_{}^{\text{L}}a={}_{}^{\text{G}}g\\ R^{T}\;\left({}_{\text{G}}^{\text{L}}q\right){}_{}^{\text{L}}m={}_{}^{\text{G}}h \end{array}\right.$$

This system, however, is overdetermined - each of the two equations provides two independent constraints on the orientation ${}_{\text{G}}^{\text{L}}q$, whereas the orientation only has three degrees of freedom. In the case when there is a disagreement between the gravitational and magnetometer readings, the system will not have a solution. The disagreement could arise from random sensor noise or unmodeled field disturbances (nongravitational accelerations or magnetic field variations). A possible solution would be to define an error metric and find the quaternion which minimizes this error. However, this could still result in disturbances in the magnetic field affecting the roll and pitch, which we are trying to avoid.

To address this problem, we present a modified system of equations. The definition of the system (but not its solution) is based on the approach presented in [26]. First, we redefine the global coordinate frame *G* to be aligned with the magnetic north. Specifically, the global frame’s *x*-axis points in the same direction as the local magnetic field (the *z*-axis remains vertical). Obviously, this global frame is only “fixed” in the case when the local magnetic field does not change its heading.

Next, we modify the system in Equation (11) so that the second equation provides only one constraint. Let ${}_{}^{\text{G}}\Pi_{zx^{+}}$ be the half-plane which contains all points that lie in the global xz-plane such that *x* is non-negative. We require that the magnetic reading, when rotated into the global frame, lies on the half-plane ${}_{}^{\text{G}}\Pi_{zx^{+}}$. Thus, we guarantee that the heading will be measured with respect to magnetic north, but do not enforce a constraint on the magnetic inclination.

(12) $$\left\{\begin{array}{c} R^{T}\;\left({}_{\text{G}}^{\text{L}}q\right){}_{}^{\text{L}}a={}_{}^{\text{G}}g\\ R^{T}\;\left({}_{\text{G}}^{\text{L}}q\right){}_{}^{\text{L}}m\in {}_{}^{\text{G}}\Pi_{zx^{+}} \end{array}\right.$$

Note that when defined in this manner, the system no longer needs a priori knowledge of the direction of the earth’s magnetic field ${}_{}^{\text{G}}h$.

In the remainder of the section, we present a novel algebraic solution to obtain ${}_{\text{G}}^{\text{L}}q$ as a function of ${}_{}^{\text{L}}a$ and ${}_{}^{\text{L}}m$. We begin by decomposing ${}_{\text{G}}^{\text{L}}q$ into two auxiliary quaternions, $q_{\text{ACC}}$ and $q_{m\text{AG}}$, such that:

(13) $${}_{\text{G}}^{\text{L}}q=q_{\text{ACC}}⊗q_{m\text{AG}}$$

and:

(14) $$R\;\left({}_{\text{G}}^{\text{L}}q\right)=R\;\left(q_{\text{ACC}}\right)R\;\left(q_{m\text{AG}}\right)$$

We further define $q_{m\text{AG}}$ to have only a single degree of freedom, by setting it to:

(15) $$q_{m\text{AG}}={\left[\begin{array}{cccc} {q_{0}}_{m\text{AG}} & 0 & 0 & {q_{3}}_{m\text{AG}} \end{array}\right]}^{T}$$

It follows from the quaternion definition in Equation (1) that $q_{m\text{AG}}$ represents a rotation around the *z*-axis only. Informally, $q_{\text{ACC}}$ rotates a vector from the sensor frame to the horizontal plane of the global frame, and $q_{m\text{AG}}$ rotates it around the *z* axis to point North. In the following subsections, we present an algebraic derivation of $q_{\text{ACC}}$ and $q_{m\text{AG}}$.

### 4.1. Quaternion from Accelerometer Readings

In this subsection, we present a derivation for the auxiliary quaternion $q_{\text{ACC}}$ as a function of ${}_{}^{\text{L}}a$. The observations of the gravity vector in the two reference frames allows us to find the quaternion that performs the transformation between the two representations. The rotation in the first equation of system Equation (12) can be re-written as:

(16) $$R\;\left({}_{\text{G}}^{\text{L}}q\right){}_{}^{\text{G}}g={}_{}^{\text{L}}a$$

and decomposed by using Equation(14) obtaining:

(17) $$R\;\left(q_{\text{ACC}}\right)R\;\left(q_{m\text{AG}}\right)\left[\begin{array}{c} 0\\ 0\\ 1 \end{array}\right]=\left[\begin{array}{c} {\text{A}}_{x}\\ {\text{A}}_{y}\\ {\text{A}}_{z} \end{array}\right]$$

The representation of the gravity vector in the global frame only has a component on the *z*-axis; therefore any rotation about this axis does not produce any change on it. Consequently, Equation (17) is equivalent to:

(18) $$R\;\left(q_{\text{ACC}}\right)\left[\begin{array}{c} 0\\ 0\\ 1 \end{array}\right]=\left[\begin{array}{c} {\text{A}}_{x}\\ {\text{A}}_{y}\\ {\text{A}}_{z} \end{array}\right]$$

expanding the multiplication we obtain the following system:

(19) $$\left\{\begin{array}{c} 2\left(q_{1_{\text{ACC}}}q_{3_{\text{ACC}}}+q_{0_{\text{ACC}}}q_{2_{\text{ACC}}}\right)={\text{A}}_{x}\\ 2\left(q_{2_{\text{ACC}}}q_{3_{\text{ACC}}}-q_{0_{\text{ACC}}}q_{1_{\text{ACC}}}\right)={\text{A}}_{y}\\ q_{0_{\text{ACC}}}^{2}-q_{1_{\text{ACC}}}^{2}-q_{2_{\text{ACC}}}^{2}+q_{3_{\text{ACC}}}^{2}={\text{A}}_{z} \end{array}\right.$$

It is clear that the above system is underdetermined and has an infinite number of solutions. This is not an unexpected result because the alignment of the gravity vector from its representation in the global frame into the local frame does not give any information about the rotation around the z-axis (yaw). Thus, such alignment can be achieved by infinite rotations with definite roll and pitch angles and arbitrary yaw. To restrict the solutions to a finite number we choose $q_{3_{\text{ACC}}}=0$ simplifying the system in Equation (19) to:

(20) $$\left\{\begin{array}{c} 2q_{0_{\text{ACC}}}q_{2_{\text{ACC}}}={\text{A}}_{x}\\ -2q_{0_{\text{ACC}}}q_{1_{\text{ACC}}}={\text{A}}_{y}\\ q_{0_{\text{ACC}}}^{2}-q_{1_{\text{ACC}}}^{2}-q_{2_{\text{ACC}}}^{2}={\text{A}}_{z} \end{array}\right.$$

The above system is fully determined; solving it results in four solutions for $q_{\text{ACC}}$. Two can be discarded since they have a negative norm. The remaining two are equivalent, with all the quaternion elements switching signs between one solution and the other. For convenience, we choose the solution with positive quaternion scalar ($q_{0}$), which corresponds to the shortest-path quaternion formulation [34]. Thus, we get:

(21) $${q_{\text{ACC}}=\left[\begin{array}{cccc} \sqrt{\frac{{\text{A}}_{z}+1}{2}} & -\frac{{\text{A}}_{y}}{\sqrt{2({\text{A}}_{z}+1)}} & \frac{{\text{A}}_{x}}{\sqrt{2({\text{A}}_{z}+1)}} & 0 \end{array}\right]}^{T}$$

The formulation in Equation (21) is valid for all values of ${\text{A}}_{z}$ except ${\text{A}}_{z}=-1$ in which it has a singularity. Further, it may arise numerical instability when in proximity of the singularity point. To address this issue we provide an alternative solution to the system in Equation (19). By simply setting $q_{2_{\text{ACC}}}=0$ instead of $q_{3_{\text{ACC}}}=0$ in Equation (19) we obtain the reduced system:

(22) $$\left\{\begin{array}{c} 2q_{1_{\text{ACC}}}q_{3_{\text{ACC}}}={\text{A}}_{x}\\ 2q_{0_{\text{ACC}}}q_{1_{\text{ACC}}}={\text{A}}_{y}\\ q_{0_{\text{ACC}}}^{2}-q_{1_{\text{ACC}}}^{2}-q_{3_{\text{ACC}}}^{2}={\text{A}}_{z} \end{array}\right.$$

which admits the following two real solutions:

(23) $${q_{\text{ACC}1}=\left[\begin{array}{cccc} -\frac{{\text{A}}_{y}}{\sqrt{2(1-{\text{A}}_{z})}} & \sqrt{\frac{1-{\text{A}}_{z}}{2}} & 0 & \frac{{\text{A}}_{x}}{\sqrt{2(1-{\text{A}}_{z})}} \end{array}\right]}^{T}$$

and

(24) $${q_{\text{ACC}2}=\left[\begin{array}{cccc} \frac{{\text{A}}_{y}}{\sqrt{2(1-{\text{A}}_{z})}} & -\sqrt{\frac{1-{\text{A}}_{z}}{2}} & 0 & -\frac{{\text{A}}_{x}}{\sqrt{2(1-{\text{A}}_{z})}} \end{array}\right]}^{T}$$

and we choose the solution in Equation (23). This formulation for $q_{\text{ACC}}$ has a singularity at ${\text{A}}_{z}=1$. Therefore, the final formulation of $q_{\text{ACC}}$ that avoids the singularity problem can be obtained by combining Equations (21) and (23):

(25) $$q_{\text{ACC}}=\left\{\begin{array}{c} \begin{array}{ccc} & {\left[\begin{array}{cccc} \sqrt{\frac{{\text{A}}_{z}+1}{2}} & -\frac{{\text{A}}_{y}}{\sqrt{2({\text{A}}_{z}+1)}} & \frac{{\text{A}}_{x}}{\sqrt{2({\text{A}}_{z}+1)}} & 0 \end{array}\right]}^{T}, & {\text{A}}_{z}\geq 0\\ & {\left[\begin{array}{cccc} -\frac{{\text{A}}_{y}}{\sqrt{2(1-{\text{A}}_{z})}} & \sqrt{\frac{1-{\text{A}}_{z}}{2}} & 0 & \frac{{\text{A}}_{x}}{\sqrt{2(1-{\text{A}}_{z})}} \end{array}\right]}^{T}, & {\text{A}}_{z}<0 \end{array} \end{array}\right.$$

Effectively, we solve the singularity problem by having two separate formulations for $q_{\text{ACC}}$ depending on the hemisphere in which *a* is pointing. Note that, defined in this manner, $q_{\text{ACC}}$ is not continuous at the ${\text{A}}_{z}=0$ point. However, we will demonstrate that this problem is resolved with the formulation of $q_{m\text{AG}}$ in the following subsection.

### 4.2. Quaternion from Magnetometer Readings

In this subsection, we present a derivation for the auxiliary quaternion $q_{m\text{AG}}$ as a function of ${}_{}^{\text{L}}m$ and $q_{\text{ACC}}$. First we use the quaternion $q_{\text{ACC}}$ to rotate the body frame magnetic field vector ${}_{}^{\text{L}}m$ into an intermediate frame whose *z*-axis is the same as the global coordinate frame with orthogonal *x*-, *y*- axes pointing in unknown directions due to the unknown yaw of $q_{\text{ACC}}$.

(26) $$R^{T}\;\left(q_{\text{ACC}}\right){}_{}^{\text{L}}m=l$$

where *l* is the rotated magnetic field vector. Next, we find the quaternion ($q_{m\text{AG}}$) that rotates the vector *l* into the vector that lies on the ${}_{}^{\text{G}}\Pi_{zx^{+}}$ of Equation (12) using the following system:

(27) $$R^{T}\;\left(q_{m\text{AG}}\right)\left[\begin{array}{c} {\text{L}}_{x}\\ {\text{L}}_{y}\\ {\text{L}}_{z} \end{array}\right]=\left[\begin{array}{c} \sqrt{\Gamma }\\ 0\\ {\text{L}}_{z} \end{array}\right]$$

where

(28) $$\Gamma ={\text{L}}_{x}^{2}+{\text{L}}_{y}^{2}$$

This quaternion performs a rotation only about the global *z*-axis by aligning the *x*-axis of the intermediate frame into the positive direction of the magnetic north pole, which coincides with the *x* direction of our global frame. This rotation will only change the heading component of the orientation without affecting the roll and pitch components. Therefore, in presence of magnetic disturbances, their influence is only limited on affecting the heading angle. The quaternion $q_{m\text{AG}}$ has the following form:

(29) $$q_{m\text{AG}}={\left[\begin{array}{cccc} q_{0_{m\text{AG}}} & 0 & 0 & q_{3_{m\text{AG}}} \end{array}\right]}^{T}$$

By reordering the system in Equation (27) and substituting $q_{m\text{AG}}$ with its components we find the following simplified system:

(30) $$\left\{\begin{array}{c} \left(q_{0_{m\text{AG}}}^{2}-q_{0_{m\text{AG}}}^{2}\right)\sqrt{\Gamma }={\text{L}}_{x}\\ 2q_{0_{m\text{AG}}}q_{3_{m\text{AG}}}\sqrt{\Gamma }={\text{L}}_{y}\\ \left(q_{0_{m\text{AG}}}^{2}+q_{3_{m\text{AG}}}^{2}\right){\text{L}}_{z}={\text{L}}_{z} \end{array}\right.$$

the solution of the above system which ensures the shortest rotation is the following:

(31) $${q_{m\text{AG}}=\left[\begin{array}{cccc} \frac{\sqrt{\Gamma +{\text{L}}_{x}\sqrt{\Gamma }}}{\sqrt{2\Gamma }} & 0 & 0 & \frac{{\text{L}}_{y}}{\sqrt{2}\sqrt{\Gamma +{\text{L}}_{x}\sqrt{\Gamma }}} \end{array}\right]}^{T}$$

It is clear from the formulation of $q_{m\text{AG}}$ that the latter quaternion incurs in a singularity state for negative ${\text{L}}_{x}$ and zero ${\text{L}}_{y}$. To avoid the singularity of $q_{m\text{AG}}$ we prevent the ***l*** vector from having negative *x*- component using the following procedure. If ${\text{L}}_{x}<0$ we rotate ***l*** of 180° around the world *z*- frame, applying the quaternion $q_{\pi }={\left[\begin{array}{cccc} 0 & 0 & 0 & 1 \end{array}\right]}^{T}$ . Finally the rotated vector is used to find $q_{m\text{A}{\text{G}}^{+}}$, which has the same form of Equation (31) and aligns *l* with the magnetic north. The sequences of rotations are summarized in the quaternion multiplications below:

(32) $$q_{m\text{A}{\text{G}}^{+}}^{*}⊗q_{\pi }^{*}⊗q_{\text{ACC}}^{*}⊗m_{q}⊗q_{\text{ACC}}⊗q_{\pi }⊗q_{m\text{A}{\text{G}}^{+}}$$

where $m_{q}$ is the local magnetic field vector written as pure quaternion and $q_{\text{ACC}}^{*}⊗m_{q}⊗q_{\text{ACC}}=l_{q}$. For the sake of simplicity we consider the quaternion product between $q_{\pi }$ and $q_{m\text{A}{\text{G}}^{+}}$ as the alternative formulation of $q_{m\text{AG}}$ in the case of ${\text{L}}_{x}<0$ as following.

(33) $$q_{m\text{AG}}=q_{\pi }⊗q_{m\text{A}{\text{G}}^{+}}$$

The result of the above multiplication is shown in Equation (34):

(34) $${q_{m\text{AG}}=\left[\begin{array}{cccc} \frac{{\text{L}}_{y}}{\sqrt{2}\sqrt{\Gamma -{\text{L}}_{x}\sqrt{\Gamma }}} & 0 & 0 & \frac{\sqrt{\Gamma -{\text{L}}_{x}\sqrt{\Gamma }}}{\sqrt{2\Gamma }} \end{array}\right]}^{T}$$

The complete formulation of $q_{m\text{AG}}$ that avoids the singularity problem discussed above, is eventually obtained by combining Equation (31) with Equation (34):

(35) $$q_{m\text{AG}}=\left\{\begin{array}{c} \begin{array}{ccc} & {\left[\begin{array}{cccc} \frac{\sqrt{\Gamma +{\text{L}}_{x}\sqrt{\Gamma }}}{\sqrt{2\Gamma }} & 0 & 0 & \frac{{\text{L}}_{y}}{\sqrt{2}\sqrt{\Gamma +{\text{L}}_{x}\sqrt{\Gamma }}} \end{array}\right]}^{T}, & {\text{L}}_{x}\geq 0\\ & {\left[\begin{array}{cccc} \frac{{\text{L}}_{y}}{\sqrt{2}\sqrt{\Gamma -{\text{L}}_{x}\sqrt{\Gamma }}} & 0 & 0 & \frac{\sqrt{\Gamma -{\text{L}}_{x}\sqrt{\Gamma }}}{\sqrt{2\Gamma }} \end{array}\right]}^{T}, & {\text{L}}_{x}<0 \end{array} \end{array}\right.$$

Finally, we can generalize the quaternion orientation of the global frame relative to the local frame as the multiplication of the two quaternions $q_{\text{ACC}}$ and $q_{m\text{AG}}$ as below.

(36) $${}_{\text{G}}^{\text{L}}q=q_{\text{ACC}}⊗q_{m\text{AG}}$$

Note that the quaternion ${}_{\text{G}}^{\text{L}}q$ does not suffer from the discontinuity problem of the yaw angle given by the switching formulation of $q_{\text{ACC}}$ of Equation (25) thanks to the multiplication with $q_{m\text{AG}}$, which performs the alignment of the intermediate frame into the global frame as previously discussed.

**Figure 1:**
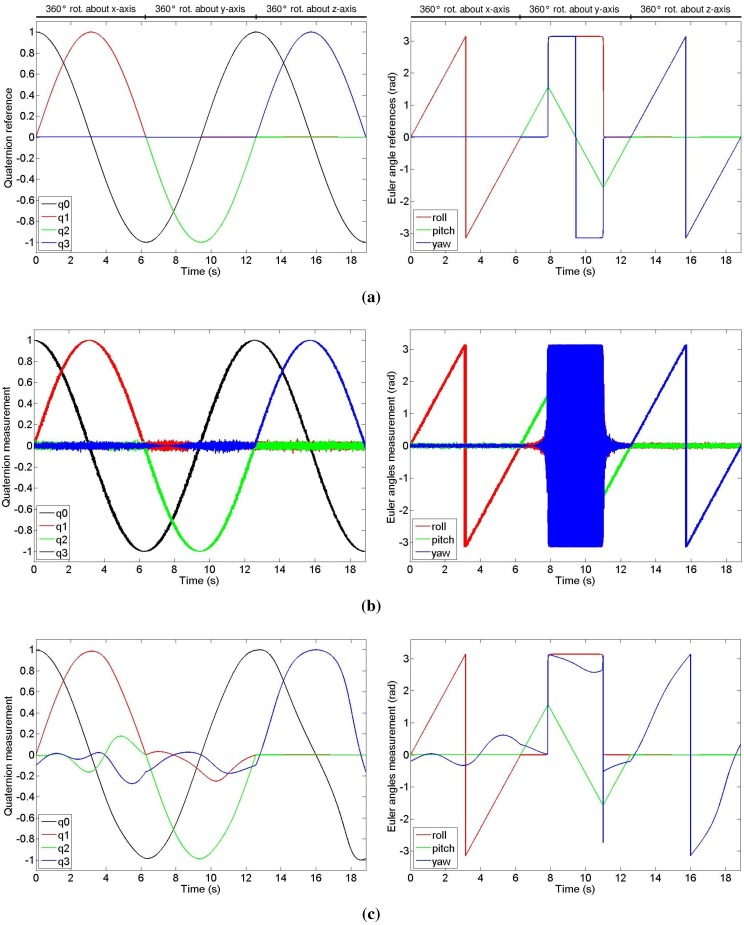
Simulated experiment showing the orientation output in quaternion form (**left**) and in Euler representation (**right**). (**a**) Orientation reference; (**b**) Orientation output of the presented method with noisy acceleration data and noise-free magnetometer data; (**c**) Orientation output of the presented method with noise-free acceleration data and magnetic readings affected by magnetic disturbances.

To test the effectiveness of our method, we implemented a MatLab simulation. The results of which are shown in Figure 1. We simulate three consecutive constant velocity (1 rad/s) rotations of 360° about each axis. First, we test the result of the quaternion solution of our proposed method by using perfect accelerometer and magnetometer data obtained by rotating the global frame vectors (gravity and magnetic field) into the local frame by using the orientation reference computed through the integration of the angular velocity vector, obtaining the same output of Figure 1a. Note, on the right side of Figure 1, that during the rotation about the *y*-axis, besides the variation of the pitch angle, we also observe an instantaneous jump of the roll and yaw angles because of the singularity configuration that affects the Euler representation. However, as shown on the left side of the figure, the quaternion representation does not incur in a singularity state; moreover, our formulation ensures the continuity of the quaternion throughout the rotation. In Figure 1b we prove that our method works with noisy acceleration data affected by Gaussian noise. When the sensor is close to some configuration where the yaw angle’s value is either π or −π, because of the noise, the yaw flips between the two values, which are two alternate representation of the same rotation. Finally, in the simulation of Figure 1c, we show the result of our orientation estimation from noise-free acceleration data and magnetometer data affected by magnetic disturbances. It is clear, from the Euler representation of the orientation, that the magnetic disturbances affect only the yaw angle.

## 5. Quaternion-Based Complementary Filter

A complementary filter uses an analysis in the frequency domain of the signals to combine them to obtain a better estimation of a particular quantity. If the signals are affected by noises with different frequency, two filters, with an appropriate bandwidth, can be applied such that the sum of the two filtered signals cover the full range of useful frequency. For attitude estimation from IMU readings, a complementary filter performs high-pass filtering on the orientation estimated from gyroscope data affected by low-frequency noise, and low-pass filter on accelerometer data affected by high-frequency noise. The fusion between the two filtered estimations will ideally obtain an all-pass and noise-free attitude estimation. The term “complementary” derives from the cut-off frequency value, which is the same for both filters. Thus, its correct value is found as a trade-off between the preserved bandwidth of each single signal.

**Figure 2:**
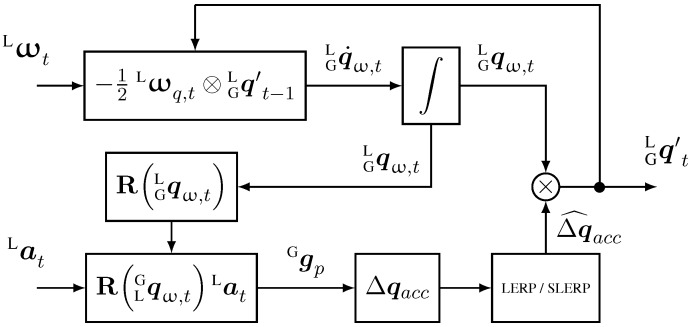
Block diagram of the complementary filter for IMU implementation (no magnetometer data).

**Figure 3:**
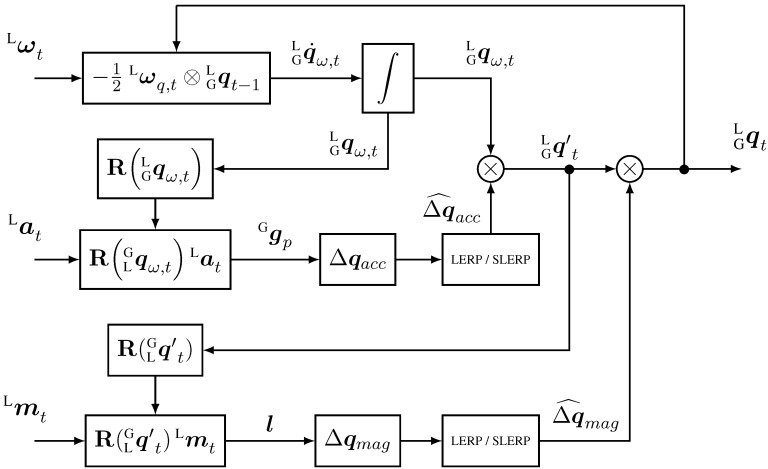
Block diagram of the complementary filter for MARG implementation (with magnetometer data).

The complementary filter we propose in this article can be used for both IMU and MARG sensors, and is described in the diagrams of Figure 2 and Figure 3. It fuses attitude estimation in quaternion form from gyroscope data, with accelerometer data in the form of a delta quaternion, which serves as correction only for roll and pitch components of the attitude maintaining the yaw estimation from the gyroscope. If magnetometer data is provided, a second step is added to the algorithm where a delta quaternion, from magnetic field readings, is derived to correct the heading of the previous estimation by performing a small rotation about the global *z*-axis in order to align the current frame with the magnetic field.

### 5.1. Prediction

In the prediction step, the angular velocity vector, measured by the tri-axis gyroscope, is used to compute a first estimation of the orientation in quaternion form. Assuming known the initial conditions, we first calculate the quaternion describing the rate of change of the orientation as a quaternion derivative, by multiplying the previous state with the angular velocity vector arranged as a pure quaternion as in Equation (6). In the literature, the quaternion derivative from an angular rate measurement is usually calculated for the forward quaternion, that is, the one representing the orientation of the local frame with respect to the global frame. Because in this article we use the inverse orientation, for the sake of clarity, we compute the quaternion derivative for our convention, starting from the formula for the forward quaternion.

It is well known that the angular rate ${}_{}^{\text{L}}\omega$ and the “forward” quaternion derivative are related by the following identity:

(37) $${{}_{\text{L}}^{\text{G}}\dot{q}}_{\omega ,t_{k}}=\frac{1}{2}\;{{}_{\text{L}}^{\text{G}}q}_{t_{k-1}}⊗{{}_{}^{\text{L}}\omega }_{q,t_{k}}$$

where ${{}_{}^{\text{L}}\omega }_{q,t_{k}}$ is the angular velocity vector arranged as pure quaternion at time instant $t_{k}$ and ${{}_{\text{L}}^{\text{G}}q}_{t_{k-1}}$ is the previous estimate of the orientation. In our case, we want to determine the derivative of the inverse unit quaternion, which is simply the conjugate of Equation (37), therefore:

$${{}_{\text{G}}^{\text{L}}\dot{q}}_{\omega ,t_{k}}={{}_{\text{L}}^{\text{G}}\dot{q}}_{\omega ,t_{k}}^{*}=\frac{1}{2}\;{\left({{}_{\text{L}}^{\text{G}}q}_{t_{k-1}}⊗{{}_{}^{\text{L}}\omega }_{q,t_{k}}\right)}^{*}=\frac{1}{2}\;{{}_{}^{\text{L}}\omega }_{q,t_{k}}^{*}⊗{{}_{\text{L}}^{\text{G}}q}_{t_{k-1}}^{*}$$

as the conjugate of the pure quaternion ${{}_{}^{\text{L}}\omega }_{q}$ is just its negative (zero scalar component), we can finally write:

(38) $${{}_{\text{G}}^{\text{L}}\dot{q}}_{\omega ,t_{k}}=-\frac{1}{2}\;{{}_{}^{\text{L}}\omega }_{q,t_{k}}⊗{{}_{\text{G}}^{\text{L}}q}_{t_{k-1}}$$

The above equation can be rewritten in matrix form as shown in Equation (39):

(39) $${{}_{\text{G}}^{\text{L}}\dot{q}}_{\omega ,t_{k}}=\Omega \;\left({{}_{}^{\text{L}}\omega }_{t_{k}}\right){{}_{\text{G}}^{\text{L}}q}_{t_{k-1}}$$

where

(40) $$\Omega \;\left({{}_{}^{\text{L}}\omega }_{t_{k}}\right)=\left[\begin{array}{cc} 0 & {{{}_{}^{\text{L}}\omega }_{t_{k}}}^{T}\\ -{{}_{}^{\text{L}}\omega }_{t_{k}} & -\left[{{}_{}^{\text{L}}\omega }_{t_{k}}\times \right]\; \end{array}\right]$$

and, regardless of the time dependence, the term $\left[{}_{}^{\text{L}}\omega \times \right]$ denotes the cross-product matrix that is associated with ${}_{}^{\text{L}}\omega$ and is equal to:

(41) $$\left[{}_{}^{\text{L}}\omega \times \right]=\left[\begin{array}{ccc} 0 & -\omega_{z} & \omega_{y}\\ \omega_{z} & 0 & -\omega_{x}\\ -\omega_{y} & \omega_{x} & 0 \end{array}\right]$$

The orientation of the global frame relative to local frame at time $t_{k}$ can be finally computed by numerically integrating the quaternion derivative using the sampling period $\Delta t=t_{k}-t_{k-1}$.

(42) $${{}_{\text{G}}^{\text{L}}q}_{\omega ,t_{k}}={{}_{\text{G}}^{\text{L}}q}_{t_{k-1}}+{{}_{\text{G}}^{\text{L}}\dot{q}}_{\omega ,t_{k}}\Delta t$$

### 5.2. Correction

The correction adopted is based on a multiplicative technique. The predicted quaternion ${{}_{\text{G}}^{\text{L}}q}_{\omega }$ (for clarity we omit the time *t*) is corrected by means of two delta quaternions as in Equation (43):

(43) $${}_{\text{G}}^{\text{L}}q={{}_{\text{G}}^{\text{L}}q}_{\omega }⊗{\hat{\Delta q}}_{\text{ACC}}⊗{\hat{\Delta q}}_{m\text{AG}}$$

Each delta quaternion is computed and filtered by the high-frequency noise independently; thus we divide the correction into two separated steps. The first step corrects the predicted quaternion only in the roll and pitch components by the application of ${\hat{\Delta q}}_{\text{ACC}}$ computed with data from the accelerometer. The second step is used only when the magnetic field readings are provided and corrects the yaw component of the quaternion orientation by applying ${\hat{\Delta q}}_{m\text{AG}}$.

#### 5.2.1. Accelerometer-Based Correction

We use the inverse predicted quaternion ${{}_{\text{L}}^{\text{G}}q}_{\omega }$ to rotate the normalized body frame gravity vector ${}_{}^{\text{L}}a$ (measured by the accelerometer) into the global frame as shown below.

(44) $$R\;\left({{}_{\text{L}}^{\text{G}}q}_{\omega }\right){}_{}^{\text{L}}a={{}_{}^{\text{G}}g}_{p}$$

obtaining the vector ${{}_{}^{\text{G}}g}_{p}$ that we call “predicted gravity”. The predicted gravity will have a small deviation from the real gravity vector; therefore, we compute the delta quaternion, $\Delta q_{\text{ACC}}$, which rotates ${}_{}^{\text{G}}g$ into ${{}_{}^{\text{G}}g}_{p}$ (because we represent the orientation of the global frame relative to the local frame), by using the following system:

(45) $$R\;\left(\Delta q_{\text{ACC}}\right)\left[\begin{array}{c} 0\\ 0\\ 1 \end{array}\right]=\left[\begin{array}{c} {\text{G}}_{x}\\ {\text{G}}_{y}\\ {\text{G}}_{z} \end{array}\right]$$

where

$${}_{}^{\text{G}}g=\left[\begin{array}{c} 0\\ 0\\ 1 \end{array}\right],\;{{}_{}^{\text{G}}g}_{p}=\left[\begin{array}{c} {\text{G}}_{x}\\ {\text{G}}_{y}\\ {\text{G}}_{z} \end{array}\right]$$

Note that the system in Equation (45) is similar to the system in Equation (18); therefore, we proceed in the same manner to find a closed-form solution. We define the component $\Delta q_{3_{\text{ACC}}}=0$ thus obtaining the following simplified system.

(46) $$\left\{\begin{array}{c} 2\Delta q_{0_{\text{ACC}}}\Delta q_{2_{\text{ACC}}}={\text{G}}_{x}\\ -2\Delta q_{0_{\text{ACC}}}\Delta q_{1_{\text{ACC}}}={\text{G}}_{y}\\ \Delta q_{0_{\text{ACC}}}^{2}-\Delta q_{1_{\text{ACC}}}^{2}-\Delta q_{2_{\text{ACC}}}^{2}={\text{G}}_{z} \end{array}\right.$$

Proceeding as for system in Equation (18) we find the following solution:

(47) $${\Delta q_{\text{ACC}}=\left[\begin{array}{cccc} \sqrt{\frac{{\text{G}}_{z}+1}{2}} & -\frac{{\text{G}}_{y}}{\sqrt{2({\text{G}}_{z}+1)}} & \frac{{\text{G}}_{x}}{\sqrt{2({\text{G}}_{z}+1)}} & 0 \end{array}\right]}^{T}$$

which is the one that ensures the shortest rotation. Although Equation (47) has a singularity for ${\text{G}}_{z}=-1$, we do not resolve it because the delta quaternion is applied at each step to correct the small deviation between the predicted gravity and the real gravity. Therefore, the value of ${\text{G}}_{z}$ will always be very close to 1. However, for a more rigorous formulation, the approach used in Equation (22) can be adopted.

As the delta quaternion is affected by the accelerometer’s high frequency noise, before applying it to the predicted quaternion, we scale it down by using an interpolation with the identity quaternion $q_{I}$. We use two different interpolation approaches based on the angle between $q_{I}$ and $\Delta q_{\text{ACC}}$. Given two quaternions *p* and *q*, the cosine of the angle Ω subtended by the arc between them is equal to the dot product of the two quaternions as shown below:

(48) $$\cos\Omega =p\cdot q=p_{0}q_{0}+p_{1}q_{1}+p_{2}q_{2}+p_{3}q_{3}$$

In our case, as the identity quaternion has the following components

(49) $${q_{I}=\left[\begin{array}{cccc} 1 & 0 & 0 & 0 \end{array}\right]}^{T}$$

the dot product is equal to the $\Delta q_{0_{\text{ACC}}}$ component of $\Delta q_{\text{ACC}}$. If $\Delta q_{0_{\text{ACC}}}>\epsilon$, where *ϵ* is a threshold value (in our case ϵ=0.9), we use a simple Linear intERPolation (LERP) as in the equation below.

(50) $${\bar{\Delta q}}_{\text{ACC}}=\left(1-\alpha \right)q_{I}+\alpha \Delta q_{\text{ACC}}$$

where α∈[0,1] is the gain that characterizes the cut-off frequency of the filter [35]. LERP does not maintain the unit norm of the delta quaternion ${\bar{\Delta q}}_{\text{ACC}}$ that is then normalized immediately after:

(51) $${\hat{\Delta q}}_{\text{ACC}}=\frac{{\bar{\Delta q}}_{\text{ACC}}}{∥{\bar{\Delta q}}_{\text{ACC}}∥}$$

**Figure 4:**
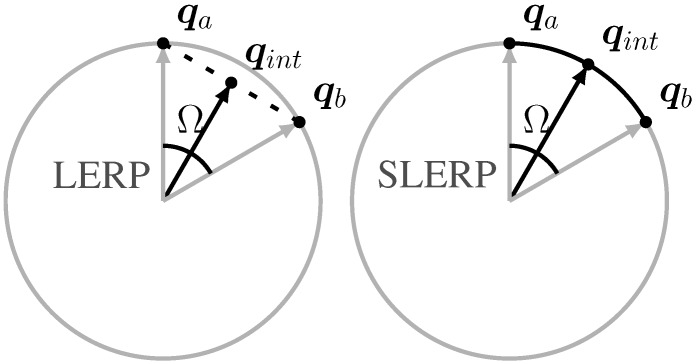
2-D representation of two different interpolation methods between quaternion ***q**_a_* and ***q**_b_* resulting in quaternion ***q**_int_*. On the left is represented the linear interpolation (LERP), whereas on the right the spherical linear interpolation (SLERP) showing the correct result.

If $\Delta q_{0_{\text{ACC}}}\leq \epsilon$, we use the Spherical Linear intERPolation (SLERP) [36]. The SLERP algorithm gives a correct evaluation of the weighted average of two points lying on a curve. In the case of quaternions, the points lie on the surface of the 4D sphere (hypersphere). A simple 2D visualization of LERP and SLERP mapping processes is shown in Figure 4. The SLERP formula we used is the following:

(52) $${\hat{\Delta q}}_{\text{ACC}}=\frac{\sin([1-\alpha ]\Omega )}{\sin\Omega }q_{I}+\frac{\sin(\alpha \Omega )}{\sin\Omega }\Delta q_{\text{ACC}}$$

The $\Delta q_{\text{ACC}}$ may have a large value when the predicted gravity has a significant deviation from the real gravity. If that condition does not occur, the delta quaternion is very small; thus, we prefer using the LERP formula because it is computationally more efficient. Finally, the quaternion estimated from gyroscope data is multiplied by the filtered delta quaternion, providing correction in the roll and pitch component, while the heading is preserved from the predicted orientation.

(53) $${}_{\text{G}}^{\text{L}}q^{\prime }={{}_{\text{G}}^{\text{L}}q}_{\omega }⊗{\hat{\Delta q}}_{\text{ACC}}$$

#### 5.2.2. Magnetometer-Based Correction

If magnetic field data is provided, a second step performs the correction on the heading component. We proceed in the same way as in the first step by computing a delta quaternion. First, we use the quaternion inverse of ${}_{\text{G}}^{\text{L}}q^{\prime }$, calculated in Equation (53), to rotate the body frame magnetic field vector ${}_{}^{\text{L}}m$ into the world frame magnetic vector.

(54) $$R\;\left({}_{\text{L}}^{\text{G}}q^{\prime }\right){}_{}^{\text{L}}m=l$$

Next, we find the delta quaternion ($\Delta q_{m\text{AG}}$), which rotates the vector *l* into the vector that lies on the xz-semiplane defined in Section 4 such that:

(55) $$R^{T}\;\left(\Delta q_{m\text{AG}}\right)\left[\begin{array}{c} {\text{L}}_{x}\\ {\text{L}}_{y}\\ {\text{L}}_{z} \end{array}\right]=\left[\begin{array}{c} \sqrt{{\text{L}}_{x}^{2}+{\text{L}}_{y}^{2}}\\ 0\\ {\text{L}}_{z} \end{array}\right]$$

This delta quaternion performs a rotation only about the global *z*-axis by aligning the global *x*-axis into the positive direction of the magnetic north pole. Further, with this formulation, such rotation does not affect the roll and pitch components even in the presence of magnetic disturbances, limiting their influence only on the heading angle. Thus, the delta quaternion $\Delta q_{m\text{AG}}$ has the following shape:

(56) $$\Delta q_{m\text{AG}}={\left[\begin{array}{cccc} \Delta q_{0_{m\text{AG}}} & 0 & 0 & \Delta q_{3_{m\text{AG}}} \end{array}\right]}^{T}$$

By rearranging the system in Equation (55) and substituting $\Delta q_{m\text{AG}}$ with its component, we find the following simplified system:

(57) $$\left\{\begin{array}{c} \left(\Delta q_{0_{m\text{AG}}}^{2}-\Delta q_{3_{m\text{AG}}}^{2}\right)\sqrt{{\text{L}}_{x}^{2}+{\text{L}}_{y}^{2}}={\text{L}}_{x}\\ 2\Delta q_{0_{m\text{AG}}}\Delta q_{3_{m\text{AG}}}\sqrt{{\text{L}}_{x}^{2}+{\text{L}}_{y}^{2}}={\text{L}}_{y}\\ \left(\Delta q_{0_{m\text{AG}}}^{2}+\Delta q_{3_{m\text{AG}}}^{2}\right){\text{L}}_{z}={\text{L}}_{z} \end{array}\right.$$

the solution of the above system that ensures the shortest rotation is the following:

(58) $$\Delta q_{m\text{AG}}={\left[\begin{array}{cccc} \frac{\sqrt{\Gamma +{\text{L}}_{x}\sqrt{\Gamma }}}{\sqrt{2\Gamma }} & 0 & 0 & \frac{{\text{L}}_{y}}{\sqrt{2(\Gamma +{\text{L}}_{x}\sqrt{\Gamma })}} \end{array}\right]}^{T}$$

with Γ as per Equation (28). The delta quaternion $\Delta q_{m\text{AG}}$ is affected by the noise of the magnetometer, which is filtered by using the procedure we adopted for $\Delta q_{\text{ACC}}$, switching between LERP and SLERP according to the same criterion. Another advantage of using two correction steps is the possibility to apply two different filtering gains. As the two delta quaternions are completely independent to each other, and each of them is related to a particular sensor (accelerometer and magnetometer), they might be affected by different frequency noise. Thus, The LERP and SLERP formulas applied to $\Delta q_{m\text{AG}}$ are the same as the ones in Equations (50) and (52), respectively, where the gain α is replaced with β, obtaining ${\hat{\Delta q}}_{m\text{AG}}$. Eventually, the delta quaternion ${\hat{\Delta q}}_{m\text{AG}}$ is applied to the quaternion in Equation (53) obtaining the final quaternion, which expresses the orientation of the global frame with respect to the local frame.

(59) $${}_{\text{G}}^{\text{L}}q={}_{\text{G}}^{\text{L}}q^{\prime }⊗{\hat{\Delta q}}_{m\text{AG}}$$

### 5.3. Adaptive Gain

A drawback of a typical implementation of the complementary filter is the constant gain that often causes inaccurate orientation estimation during highly dynamic motion. When the vehicle moves with high acceleration, the magnitude and direction of the total measured acceleration vector are different from gravity; therefore the attitude is evaluated using a false reference, resulting in significant, possibly critical errors. However, the gyroscope readings are not affected by linear acceleration, thus they can still be used to compute a relatively accurate orientation estimation that, under this condition, should be treated as the main source of the estimate. A constant gain fusion algorithm cannot overcome the aforementioned problem if the optimal gain has been evaluated for static conditions. In this paper we address this issue adopting an adaptive gain whose strategy is slightly different from the switching approach proposed in [30].

**Figure 5:**
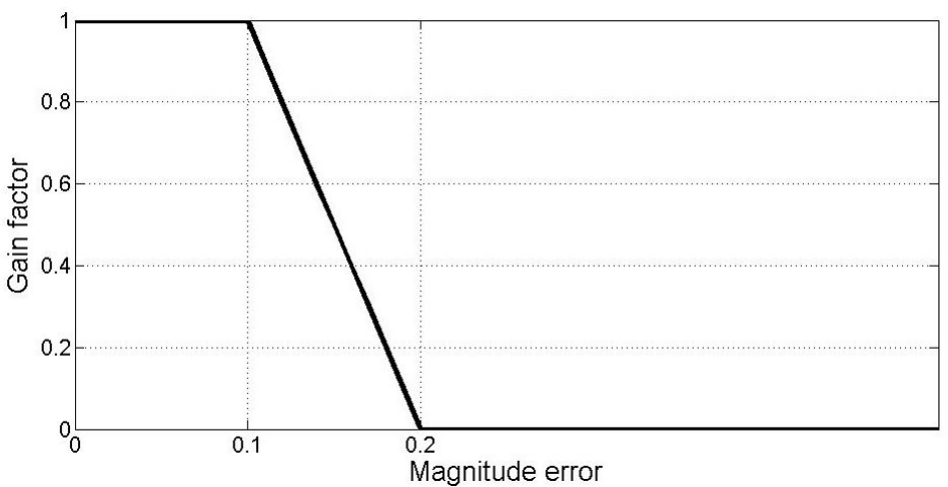
The gain factor function used in the adaptive gain to reduce the attitude error deriving from the external linear acceleration because of vehicle motion.

First we define the magnitude error $e_{m}$ as in the following equation:

(60) $$e_{m}=\frac{\left|∥{}_{}^{\text{L}}\tilde{a}∥-\text{G}\right|}{\text{G}}$$

where $∥{}_{}^{\text{L}}\tilde{a}∥$ is the norm of the measured local frame acceleration vector before normalization and *g* = 9.81 m/s${}^{2}$. Given Equations (50) and (52), we make the filtering gain α function of the magnitude error $e_{m}$ through the gain factor *f*, meaning:

(61) $$\alpha =\alpha \left(e_{m}\right)=\bar{\alpha }f\left(e_{m}\right)$$

where $\bar{\alpha }$ is the constant value that gives the best filtering result in static conditions and $f(e_{m})$ is what we call the “gain factor”, which is a piecewise continuous function of the magnitude error as shown in Figure 5. The gain factor is constant and equal to 1 when the magnitude of the nongravitational acceleration is not high enough to overcome the acceleration of gravity and the value of the error magnitude does not reach the first threshold. If the nongravitational acceleration rises and the error magnitude exceeds that first threshold, the gain factor decreases linearly with the increase of the magnitude error until reaching zero for error magnitude equal to the second threshold and over. Empirically, we found that the two thresholds’ values that give the best results are respectively 0.1 and 0.2.

In this article we do not address the problem of the centripetal acceleration; however, if conditions warrant, one can easily add the centripetal force model used in [21].

### 5.4. Filter Initialization and Bias Estimation

The complementary filter proposed in this article is initialized with the orientation quaternion calculated using the procedure explained in Section 4. The values of the current body-frame acceleration and magnetic field vectors are used to produce the quaternion representing the initial orientation of the rigid body in any configuration. Therefore, for the initialization, the filter does not need any assumption and it is performed in one single step.

Before using the angular velocity vector for the quaternion prediction, we correct it for the bias. The bias of a sensor’s reading is a slow-varying signal considered as low frequency noise. Therefore, we adopt a low-pass filter to separate the bias from the actual angular velocity reading. To avoid filtering useful information, the low-pass filtering is applied only when the sensor is in a steady-state condition that is previously checked. If the sensor is in the steady-state condition, the bias is updated, otherwise it is assumed to be equal to the previous step value. The estimated bias is then subtracted from the gyroscope reading obtaining a bias-free angular velocity measurement. The low-pass filter cut-off frequency can be selected by the user as well as parameters for the steady-state condition, to apply the bias-estimation to different sensors.

## 6. Experiments

In this section, we evaluate the performances of the proposed complementary filter under different conditions and we compare it against other common estimation methods. At first we evaluate the overall performances during MAV flights, then we evaluate and compare the behavior of the different methods under, respectively, magnetic disturbances and high nongravitational acceleration.

### 6.1. MAV Flight Experiment

In the first experiment, we evaluated the accuracy of the orientation estimation algorithm using publicly-available MAV datasets [37] with ground-truth orientation in Euler-angles form from a motion-capture system.

**Figure 6:**
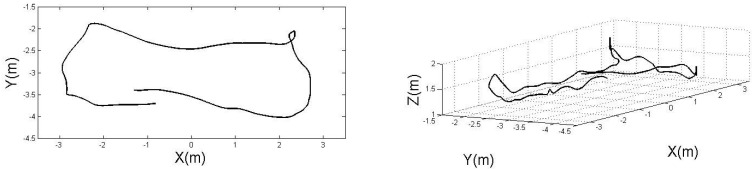
2D top view (**left**) and 3D side view (**right**) of the trajectory traveled by the quadrotor during the “1LoopDown” experiment.

**Figure 7:**
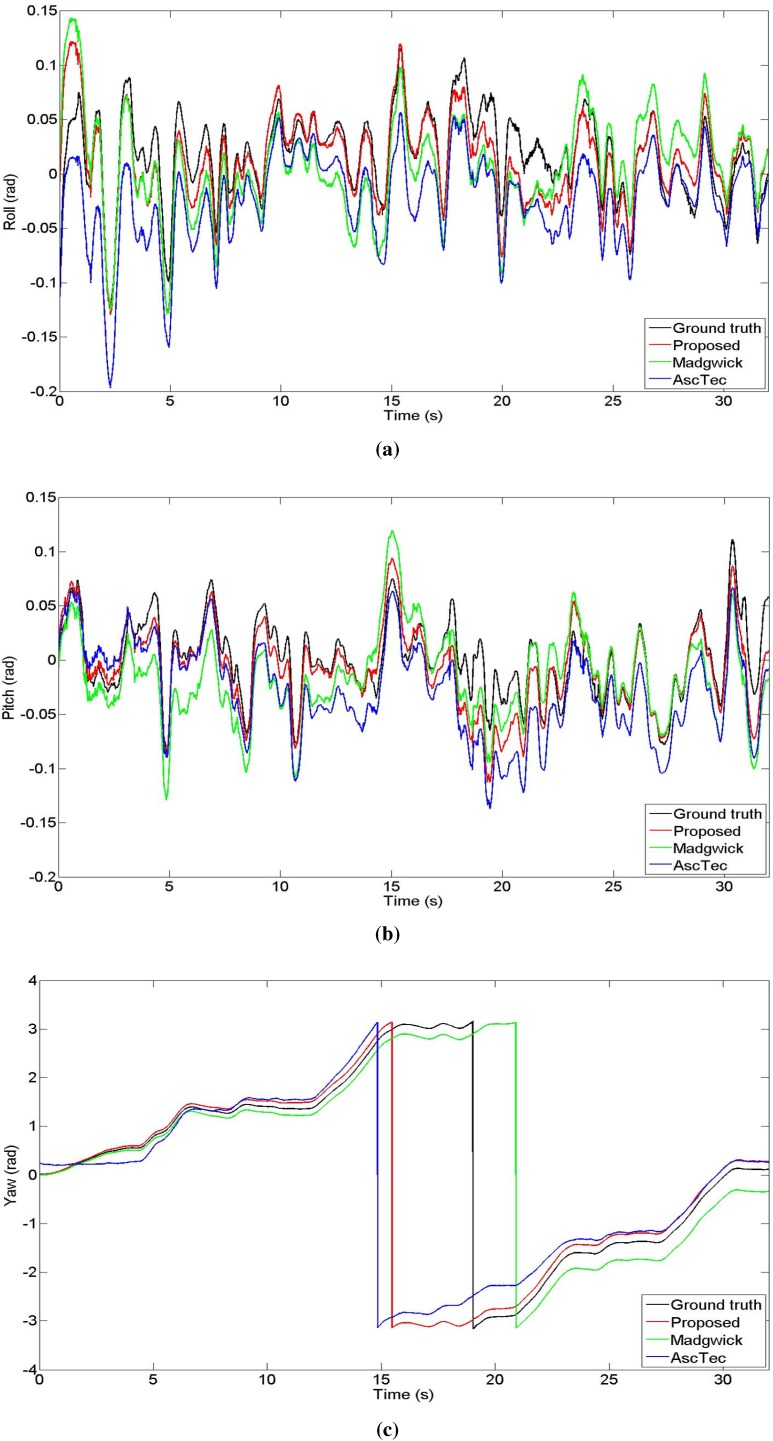
Comparison of the orientation Euler angles output of the three methods during the “1LoopDown” experiment. (**a**) Roll; (**b**) Pitch; (**c**) Yaw.

The datasets are recorded using an AscTec “Pelican” quadrotor, flying in an indoor environment of size 10 m×10 m×10 m, equipped with eight Vicon cameras, performing 1, 2, and 3 loops, respectively. Figure 6 shows the trajectory traveled by the quadrotor during the “1loop” experiment, tracked by the motion capture system. The datasets include acceleration and angular velocity readings from the IMU, and no magnetic field data. We compare our method against the orientation estimation proposed by Madgwick *et al*. in [26], the quaternion-based EKF proposed by Sabatini [24], and the algorithm embedded in the low-level processor of the AscTec quadrotor, whose output, in Euler angles form, is provided in the datasets. Table 1, Table 2 and Table 3 show the Root Mean Square (RMS) error for roll, pitch, and yaw angles, respectively, in the three datasets, proving that our algorithm outperforms the other methods for all the sets. Note that the proposed method and one of the algorithms against which we compare it, by Madgwick *et al*. are constant gain filters. Therefore, their performance will vary based on the value of the chosen gain. To obtain a fair qualitative comparison, we chose the gains that minimize the RMS error of the estimation for each method. Figure 7 shows the orientation in Euler angles for the four different algorithms using the data from the “1LoopDown” dataset. Note that as the magnetic field readings are not provided, no correction is available for the yaw component. However, enabling our bias estimation allows a significant reduction of the drift error.

**Table 1:** RMS roll angle error [radians].

Dataset	Proposed	Madgwick	AscTec	EKF
1LoopDown	**0.0233**	0.0370	0.0464	0.0287
2LoopsDown	**0.0292**	0.0470	0.0338	0.0314
3LoopsDown	**0.0277**	0.0405	0.0315	0.0331

**Table 2:** RMS pitch angle error [radians].

Dataset	Proposed	Madgwick	AscTec	EKF
1LoopDown	**0.0209**	0.0336	0.0369	0.0284
2LoopsDown	**0.0223**	0.0369	0.0313	0.0384
3LoopsDown	**0.0202**	0.0360	0.0329	0.0392

**Table 3:** RMS yaw angle error [radians].

Dataset	Proposed	Madgwick	AscTec	EKF
1LoopDown	**0.1429**	0.2543	0.3388	0.1888
2LoopsDown	**0.1309**	0.9229	0.3182	0.3345
3LoopsDown	**0.2890**	1.3327	0.3255	0.3545

### 6.2. Magnetic Disturbances

**Figure 8:**
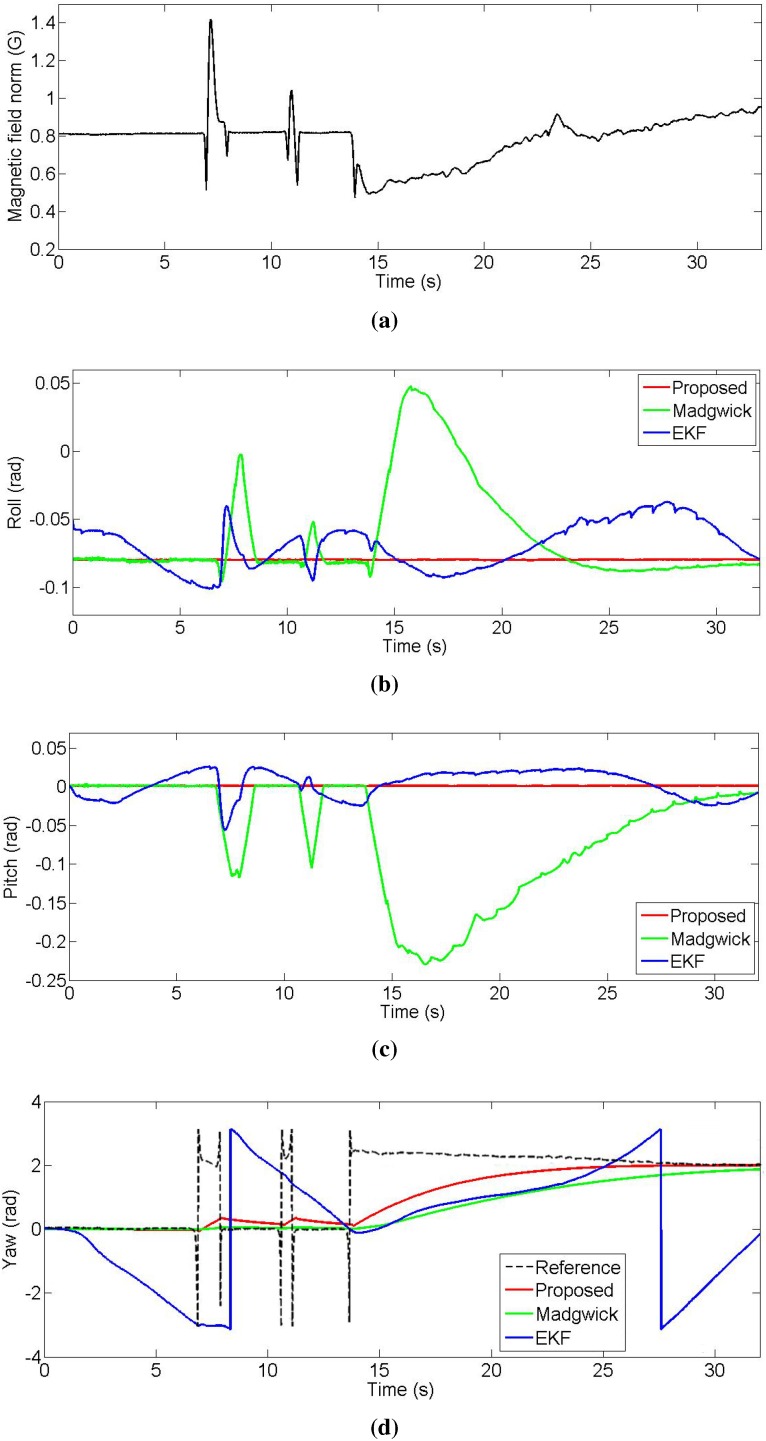
Comparison of the different estimation methods in the presence of magnetic disturbances. (**a**) Norm of the measured magnetic field vector; (**b**) Roll; (**c**) Pitch; (**d**) Yaw.

In the second experiment, we used a Phidgets Spatial 3/3/3 sensor. It provides acceleration, angular rate, and magnetic field strength measurements in three axes. In this case, we computed the orientation using acceleration as well as magnetic field data. We held the sensor in a steady state while reading the output of our complementary filter, the EKF, and the filter by Madgwick *et al*. We induced magnetic disturbances by approaching a magnet to the sensor and repeated this action thrice. The first two times we applied the magnetic disturbance only for a couple of seconds, whereas for the third time, we retained the disturbance until the end of the experiment. Figure 8 shows the results of the experiment. The roll and pitch angles from our complementary filter are completely immune to magnetic disturbances. The roll and pitch from the Madgwick filter are affected by the magnetic disturbances when we approach the magnet to the sensor, converging back to the right value when the disturbance is removed. When we induce the disturbance the third time, the roll and pitch of the Madgwick filter present a similar behavior, but with a slower convergence, showing that the roll and pitch angles are only immune to constant magnetic flux. The EKF has a random behavior because it is already affected by the magnetic flux present in the room caused by electric devices, as a consequence of the coupled nature of the acceleration and magnetic field correction. In fact, although the norm of the measured magnetic field is constant at the beginning of the experiment, its value is different from the norm of the reference magnetic field vector (about 0.54 Gauss in this area), vector that is needed as input to the EKF. At the bottom of Figure 8 we show the yaw angles output of the three algorithms, during the same experiment, compared against the reference yaw angle extracted from the magnetic field vector. Unlike the Madgwick filter, the proposed complementary filter has the advantage of having a dedicated gain (β) to filter the magnetic field measurement noise. This allows us to change the yaw sensitivity to the reference variations. For this experiment, we chose the gain value that gives the best trade-off between the error because of the magnetic disturbances and the rate of the convergence. Note that although the Madgwick filter can vary its magnetic sensitivity by changing the filtering gain, this would also cause a performance degradation in the estimation of the roll and pitch components because only one gain controls the cut-off frequency of the signals coming from two different sensors. In the case of the EKF, when the measurement covariance comes directly from the sensor’s standard deviation, the yaw output is very sensitive to the magnetic disturbances as can be seen in the figure. However, it is well known that the measurement covariance can be changed to make the algorithm less sensitive to the selected measurement.

### 6.3. High Nongravitational Acceleration

In this experiment, we analyze the efficiency of our complementary filter adopting the adaptive gain introduced in Section 5.3 under the condition of high nongravitational acceleration. We used the previously described Phidgets Spatial 3/3/3 sensor, attached to a slider, free to translate over a camera rail track, such that the sensor’s *x*-axis is pointing along the direction of the translation. This setup allows us to create accelerated motion even as maintaining the IMU on the same plane throughout the experiment with constant zero attitude. We induced a high linear acceleration on the x−body axis by moving the slider abruptly back and forth. Note that because the sensor moves in the horizontal plane, the inertial forces measured by the IMU are completely decoupled: gravitational acceleration on the *z*-axis, and total body motion acceleration on the *x*-axis.

**Figure 9:**
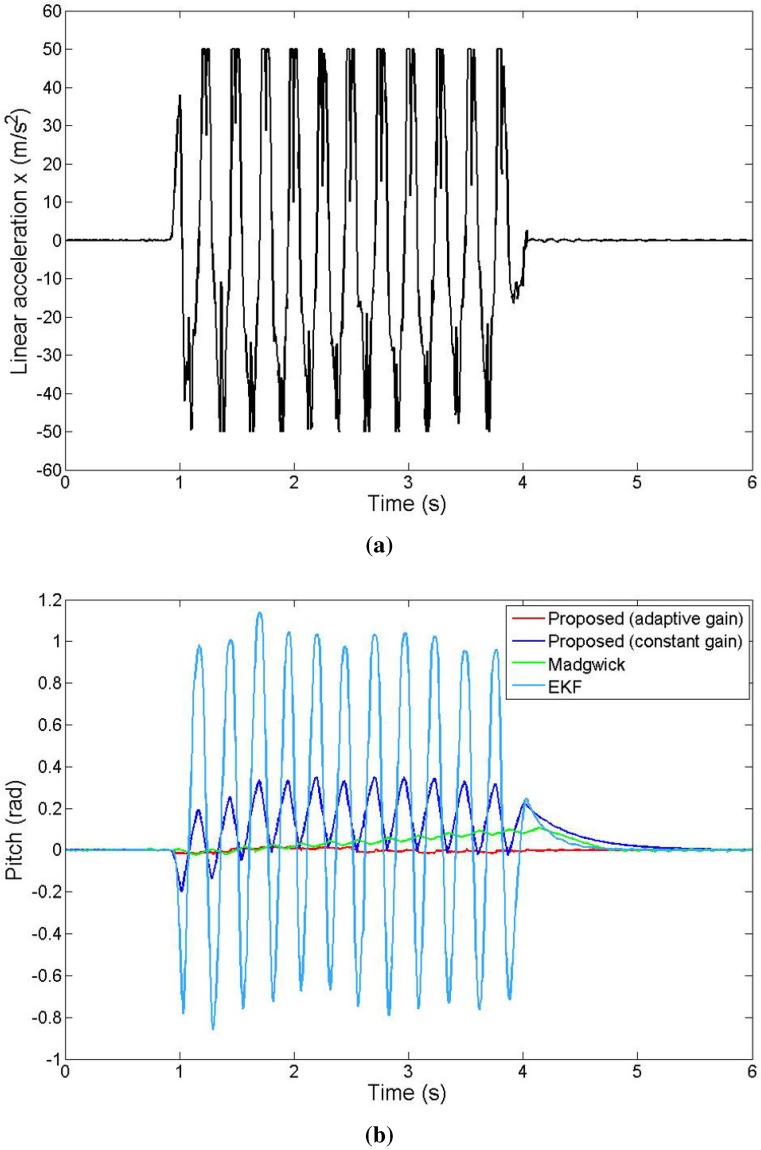
Pitch estimation under the condition of high nongravitational acceleration; (**a**) Non gravitational acceleration applied during the experiment; (**b**) Pitch angle output of the different algorithms

We evaluate the improvement of our method from the addition of the adaptive gain. Figure 9 shows the pitch angle output of the proposed algorithm with constant and adaptive gain, as well as the pitch angle output of the EKF and the Madgwick filter. The acceleration because of the rigid-body motion has a varying value reaching a maximum of 50 m/s${}^{2}$. This causes an error of the output of the constant gain complementary filter to reach 0.3 radians, whereas the output of the adaptive gain filter has an error which never exceeds 0.02 radians. Moreover, the proposed adaptive gain complementary filter clearly outperforms the other two methods that do not have a solution to reduce the attitude error during accelerated motion.

Ultimately, we analyzed the computational time of the three methods. We profiled a reasonably optimized C++ implementation of all the algorithms running on an Intel core I7, 3.6 GHz processor. Table 4 summarizes the results for the average execution time of a prediction-correction update cycle. The results demonstrate that the proposed complementary filter and the Madgwick algorithm have very similar processing times suitable for small embedded processors, whereas the EKF has a runtime almost six times higher. Note that given the higher standard deviation of the average time measurement relative to the Madgwick filter, we can consider negligible the small difference of 0.14 μs between the two faster approaches.

**Table 4:** Computational time of estimation algorithms.

Algorithm	Average Time (μs)	Standard Deviation (μs)
Proposed	1.4243	0.4761
Madgwick	1.2839	0.7101
EKF	7.0408	0.2342

## 7. Conclusions

In this article we presented a novel deterministic solution to the Whaba’s problem to find the orientation in quaternion form given two pairs of vector observations from inertial sensors. We are able to find the orientation of the global frame with respect to the local frame without leading to ambiguous results or singularity problems. The subdivision of the quaternion in two parts makes the roll and pitch components immune to magnetic distortions. Further, the procedure adopted to find the heading component of the orientation eliminates the need for a direction magnetic field to be predefined. We demonstrated analytically and empirically the validity of the proposed method.

We also presented a novel quaternion-based complementary filter, which fuses together gyroscope data with accelerometer and magnetic field data. The predicted quaternion is calculated by using the well-known linear formulation of the quaternion rate of change from the gyroscope’s angular rate. The correction is applied by means of two delta quaternions that are computed using an approach based on the method described in Section 4. The presented algorithm offers several advantages over other implementations:

- Fast initialization in quaternion form allowing any starting configuration.
- Fast convergence of the orientation quaternion because of the algebraic formulation of the delta quaternions.
- Two different gains to separately filter acceleration and magnetic field noises.
- Magnetic distortion compensation that involves a two-fold advantage: it avoids the impact of the magnetic disturbances on the roll and pitch components of the orientation when the sensor is surrounded by unwanted magnetic flux and eliminates the need of a predefined magnetic field direction.

Moreover, our algorithm does not compute any matrix inversion or matrix multiplication, maintaining a low computational cost making it suitable for onboard implementation.

We evaluated our complementary filter using publicly available data of a micro quadrotor helicopter flying in an indoor environment. We compared our results against other algorithms, showing better performances of our method.
